# Secondary TEVAR After Frozen Elephant Trunk: Indications and Timing

**DOI:** 10.3390/jcm15218585

**Published:** 2026-11-04

**Authors:** Dimitrios E. Magouliotis, Beatrice Bacchi, Arian Arjomandi Rad, Andrew Xanthopoulos, Tulio Caldonazo, Michele D’Alonzo, Massimo Baudo, Francesco Cabrucci, Francesca Maria di Muro

**Affiliations:** 1 Department of Cardiac Surgery Research, Lankenau Institute for Medical Research, Main Line Health, Wynnewood, PA 19096, USA; massimo.baudo@icloud.com; 2 NYU Grossman School of Medicine, NYU Langone Health, New York, NY 10016, USA; beatricebacc@gmail.com; 3 Department of Cardiothoracic Surgery, John Radcliffe Hospital, Oxford University Hospitals NHS Foundation Trust, Oxford OX3 9DU, UK; arian.arjomandirad@gmail.com; 4 Department of Cardiology, University Hospital of Larissa, 41110 Larissa, Greece; anxanthopoulos@med.uth.gr; 5 Department of Cardiothoracic Surgery, Jena University Hospital, 07747 Jena, Germany; tulio.caldonazo@med.uni-jena.de; 6 Cardiac Surgery Unit, Poliambulanza Foundation Hospital, Via Bissolati 57, 25124 Brescia, Italy; 7 Department of Cardiovascular and Thoracic Surgery, Lenox Hill Hospital, Northwell Health, New York, NY 10075, USA; francesco.cabrucci.6@gmail.com; 8 Department of Medicine, Surgery and Dentistry, University of Salerno, Via Giovanni Paolo II 132, 84084 Salerno, Italy; fdimuro94@gmail.com

**Keywords:** frozen elephant trunk, thoracic endovascular aortic repair, aortic dissection, aortic remodeling, false lumen, distal stent graft-induced new entry, aortic arch surgery, hybrid aortic repair

## Abstract

Frozen elephant trunk (FET) stabilizes the arch and proximal descending aorta, but complete distal remodeling is achieved in only a subset of patients with aortic dissection. Persistent false lumen perfusion, aortic growth, and distal stent graft-induced new entry lead to secondary thoracic endovascular aortic repair (TEVAR), and reported distal reintervention rates range from about 13% at 5 years in pooled data to 33% in a center with routine staging. This narrative review examines the indications for and timing of secondary TEVAR after FET. PubMed was searched to September 2026, and 97 publications were included. Evidence obtained in patients treated with FET is distinguished from evidence extrapolated from primary type B dissection and from the authors’ interpretation. We summarize the predictors of incomplete remodeling, including stent graft length, residual re-entry location, chronicity, aortic diameter at the distal stent level, early remodeling trajectory, and heritable aortopathy, and we contrast planned completion with surveillance-based reactive TEVAR, including technical aspects and spinal cord protection. Direct evidence on timing after FET is limited to retrospective series and one pilot randomized trial of TEVAR 1 to 3 months after type I repair; most data on the time-dependent loss of flap plasticity come from primary type B dissection. No universal timing rule is supported. We propose a phenotype-driven approach, not yet validated, in which timing is referenced to dissection onset and individualized to aortic anatomy, disease progression, and clinical condition.

## 1. Introduction

The frozen elephant trunk (FET) technique descends from the conventional elephant trunk described by Borst in 1983 and from the stented graft implanting method introduced by Kato and colleagues in 1996 [1,2]. Karck and coworkers established the hybrid prosthesis in Europe, and Sun and colleagues demonstrated in parallel that total arch replacement with a stented elephant trunk could be performed with low early mortality in acute type A dissection [3,4]. The operation is now the reference treatment for arch disease that extends into the proximal descending aorta and is endorsed by the European Association for Cardio-Thoracic Surgery (EACTS) position paper, the EACTS and European Society for Vascular Surgery (ESVS) expert consensus, and the 2024 EACTS and Society of Thoracic Surgeons (STS) guidelines [5,6,7]. The appeal is twofold: a single operation replaces the arch and stabilizes the proximal descending aorta, and the stented segment creates a durable proximal landing zone for any later endovascular or open extension [8,9]. The second benefit matters because the first is often incomplete. Reported distal reintervention rates vary with treatment strategy, device, anastomotic zone, case mix, and follow-up. A meta-analysis of 28 studies including 11,292 patients reported freedom from distal reintervention of 93.9%, 87.4%, and 81.5% at 1, 5, and 10 years [10]. In Freiburg, where a zone 2 anastomosis with a 100 mm stent graft and planned staging were routine, 33% of 107 FET patients underwent aortic reintervention, and the cumulative risk reached 31%, 49%, and 64% at 12, 24, and 36 months [11]; distal aortic failure occurred in 47.3% of 186 dissection patients in an overlapping cohort from the same center [12]. In the Bologna and Hannover experience, 86 patients (23.1% of hospital survivors) required an additional procedure, which was endovascular in 61 (16.3%) [13]. Distal stent graft-induced new entry (dSINE) has been reported in 2% of patients in pooled data, in 13% in a two-center series, and in 51% of patients operated on for chronic dissection [14,15,16], and post-dissection aneurysm formation after surgery for acute type A dissection has a pooled incidence of 10.24% [17].

For a substantial subset of patients, the clinical question has therefore shifted from whether the downstream aorta will need attention to when and how it should be treated. Two strategies dominate practice. The first treats FET as the first step of a planned two-stage repair and schedules completion of thoracic endovascular aortic repair (TEVAR) within weeks to months, before the dissection flap stiffens and before the aorta enlarges. The second relies on surveillance and deploys TEVAR only when imaging shows growth, persistent false lumen perfusion, dSINE, or malperfusion. Both approaches are supported mainly by retrospective series. A pilot randomized trial of TEVAR 1 to 3 months after surgery for type I dissection has been reported, and a multicenter randomized trial of early endovascular repair after FET is under way [18,19], but current guidelines do not define a timing rule [7,20]. This narrative review summarizes the anatomic and procedural predictors of incomplete remodeling after FET, the indications for secondary TEVAR, the evidence behind planned and reactive timing, the technical demands of landing within a hybrid prosthesis, spinal cord protection, and the overlap with branched arch endografts. Throughout, we separate three levels of support: evidence obtained in patients treated with FET, evidence extrapolated from primary TEVAR for type B dissection or from open staged repair, and the authors’ interpretation. We close with a phenotype-driven approach that is proposed for prospective testing, not as a validated algorithm.

## 2. Literature Search, Study Selection, and Terminology

We searched PubMed (MEDLINE) on 23 and 24 September 2026 without date or language restrictions. Nineteen search strings combined terms for the frozen elephant trunk and hybrid prostheses with terms for thoracic endovascular aortic repair, completion, secondary intervention, reintervention, distal stent graft-induced new entry, aortic remodeling, false lumen, spinal cord ischemia, staged repair, branched arch endografts, and timing; the complete strings and the number of records returned by each are listed in Supplementary Table S1. These searches returned 1432 records before removal of duplicates. Targeted searches identified guidelines, consensus documents, reporting standards, and randomized trials, and the reference lists of relevant reviews were screened by hand. Other databases were not searched.

Titles and abstracts were screened, and the full texts of potentially relevant articles were reviewed against predefined criteria. We included guidelines and consensus documents; systematic reviews and meta-analyses; randomized trials and trial protocols; registries and observational series reporting at least 10 patients with secondary endovascular treatment after FET; studies of predictors of distal remodeling, dSINE, or spinal cord ischemia after FET or TEVAR; and studies of TEVAR timing or technique in type B dissection when they informed the questions of this review. We excluded case reports, series of fewer than 10 patients reporting outcomes of secondary treatment, conference abstracts, and articles not published in English. In total, 97 publications were included: 7 guidelines, consensus documents, and reporting standards; 11 systematic reviews and meta-analyses; 4 randomized trials or trial protocols; 59 observational cohorts, registries, and prospective device studies; and 16 narrative reviews, technical descriptions, and experimental or computational studies. Cohorts from the same institution were treated as potentially overlapping. Consistent with the narrative design, screening was not performed in duplicate, and no formal risk-of-bias assessment or quantitative synthesis was performed.

To separate evidence from interpretation, each statement on timing is attributed to one of three sources: direct evidence from patients treated with FET, evidence extrapolated from primary TEVAR for type B dissection or from open staged repair, and the authors’ interpretation; this attribution is summarized for the main elements of the proposed approach in Section 11. Chronicity is counted from symptom onset according to the Society for Vascular Surgery (SVS) and Society of Thoracic Surgeons (STS) reporting standards: hyperacute, less than 24 h; acute, 1 to 14 days; subacute, 15 to 90 days; and chronic, more than 90 days [21]. Intervals are stated relative to dissection onset or to the index FET, and the reference point is named each time. After FET for acute dissection, which is usually performed within days of onset, the two intervals differ by days; after FET for chronic dissection, they differ by months or years, and the remodeling potential of the flap is set by the age of the dissection rather than by the interval after FET. Aortic zones follow the SVS and STS map, in which zones 3 to 5 comprise the descending thoracic aorta and zones 6 to 8 the paravisceral aorta from the celiac trunk to the renal arteries [21].

## 3. The Frozen Elephant Trunk as a Staging Platform

### 3.1. Device Design and the Concept of Staged Aortic Repair

Contemporary FET prostheses combine a proximal polyester graft, straight or branched, with a distal self-expanding stent graft that is deployed antegrade into the descending aorta during circulatory arrest [22]. Platforms differ in stent length, radial force, distal-end configuration, and collar design, and stent lengths used in published series range from 60 to 180 mm [23,24,25]. The E-vita Open NEO, for example, is available with stented segments of 120 to 180 mm and in branched, straight, and trifurcated versions [25]. These differences affect the distal aorta. In a bicentric comparison of 88 acute dissections, dSINE rates were similar with the Thoraflex Hybrid and E-vita Open devices (16% versus 18%), but secondary endovascular interventions were more frequent with Thoraflex (22% versus 0%), and false lumen thrombosis at the stent level trended lower (74% versus 95%), a difference the authors suggested might reflect longer coverage of the descending aorta with the E-vita design and more frequent zone 3 implantation [26]. Bench testing showed that the distal end of the Thoraflex prosthesis stiffens sharply with small displacements, whereas the E-vita prosthesis behaves with nearly constant stiffness [15]. Reviews of device-specific data conclude that prosthesis choice, diameter, and length all influence the need for reintervention [27].

**Figure 1:**
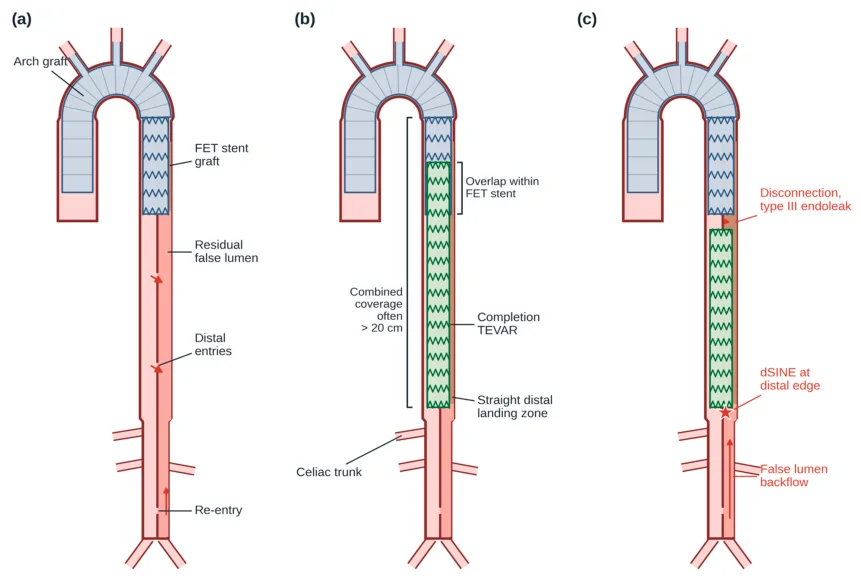
Anatomy of completed thoracic endovascular aortic repair (TEVAR) after the frozen elephant trunk (FET). (**a**) FET in place after total arch replacement, with the stent graft ending in the proximal to mid descending aorta and residual dissection distally, including false lumen perfusion through distal entries. (**b**) Completion TEVAR landing proximally within the FET stent graft with generous overlap and distally in a straight segment above the celiac trunk; the combined covered length frequently exceeds 20 cm. (**c**) Principal failure modes: distal stent graft-induced new entry (dSINE) at the distal edge, persistent false lumen backflow from abdominal re-entries, and disconnection between the FET and the TEVAR stent graft with type III endoleak. Schematic, not to scale.

These observations have changed how aortic surgeons view the operation. FET is increasingly framed as the first step of lifetime aortic management rather than as a definitive repair, with a planned or anticipated second endovascular stage and, in a minority, a third open or branched stage [8]. The stent graft provides an ideal proximal landing zone for TEVAR, and when endovascular extension fails, open thoracoabdominal replacement is simplified because the proximal anastomosis has moved distally [9]. The practical consequence is that decisions made at the index operation, including the proximal zone, stent length, and distal landing level, define the geometry, the spinal cord reserve, and the landing options available for the second stage (Figure 1).

### 3.2. Proximal Zone, Stent Length, and Distal Landing Level

Moving the distal anastomosis to zone 2 shortens circulatory arrest, simplifies the operation, and reduces recurrent laryngeal nerve injury, but it also moves the distal end of the stent graft proximally unless a longer stent is used. In 232 patients treated between 2009 and 2020, secondary aortic interventions were most frequent when a zone 2 anastomosis was combined with a 100 mm stent graft whose distal end lay at the level of the second or third thoracic vertebra, and they were less frequent when the distal end reached below the fourth or fifth thoracic vertebra; no association was found between the anastomotic zone and spinal cord injury [23]. In 191 patients undergoing zone 0 arch repair for acute type A dissection, a 150 mm stent positioned around the eighth thoracic vertebra was associated with numerically lower residual distal malperfusion (2.7% versus 6.5%, *p* = 0.62) and new spinal cord ischemia (0% versus 1.9%, *p* = 1.00) than shorter stents, in nonrandomized groups of 37 and 154 patients [24]. A 150 mm stent produced better thoracic remodeling than a 100 mm stent in 158 acute dissections, with spinal cord injury in 2.2% and 5.3% (not significant) [28], and a series of 51 zone 2 operations with 15 cm stents reported no spinal cord ischemia [29]. Pooled data point in the other direction: in a meta-analysis of 3154 patients, spinal cord ischemia occurred in 11.6% after stents of at least 15 cm or coverage to the eighth thoracic vertebra or beyond, compared with 2.5% after 10 cm stents [30], and a distal stent end below the ninth thoracic vertebra was an independent risk factor for spinal cord injury in 224 patients (odds ratio 15.2) [31]. In a smaller cohort, a zone 3 proximal landing produced higher complete false lumen thrombosis at 24 months than zone 2 (77% versus 60%) [32]. Zone 2 arch replacement is now also used deliberately as preparation for TEVAR: among 142 patients, 45.1% received a concomitant FET, and all five cases of spinal cord ischemia, all transient, occurred in that subgroup [33]. The trade-off is explicit. A short stent in zone 2 increases the likelihood of a second stage, whereas a long stent improves thoracic remodeling at the cost of segmental arteries, spinal cord reserve, and native landing length above the celiac trunk.

## 4. Distal Aortic Fate After the Frozen Elephant Trunk

### 4.1. Defining Remodeling and Failure

Remodeling definitions vary widely and complicate comparisons. Dohle and colleagues used volumetric thresholds, classifying remodeling as positive when the true lumen increased by more than 10% with a stable aortic lumen, or when the aortic lumen decreased by more than 10% with a stable true lumen, and as negative for all other changes outside a 10% band [34]. Other groups rely on maximal diameters, false lumen thrombosis status, or composite definitions. Complete thoracic remodeling has been defined as thoracic false lumen thrombosis with a false lumen diameter below 10 mm down to the tenth thoracic vertebra [35], and distal aortic failure as distal reintervention, a diameter of at least 6 cm, growth of more than 5 mm within 6 months, dSINE, or aortic-related death [12]. The ARCH Academic Research Consortium has now published standardized endpoint definitions for thoracic aortic repair trials, and their adoption would make future studies of secondary TEVAR comparable [36]. Chronicity matters for interpretation. After FET for acute dissection, the interval after the index operation approximates the interval from onset, but chronic dissections account for a large share of FET cohorts, 95 of 186 patients in one series [12], and in these patients a given interval after FET corresponds to a much older dissection (Section 2).

### 4.2. Segmental Remodeling Patterns

Remodeling after FET is reliable at the stent level and progressively less reliable downstream. In 70 patients with 1-year imaging, positive or stable remodeling in acute dissection occurred in 90% of segments along the stent graft, 65% from the stent to the celiac trunk, and 58% below the celiac trunk; in chronic dissection the corresponding rates were 100%, 86%, and 77% at 1 year but fell to 75%, 44%, and 38% at later follow-up, and negative remodeling in two or more segments occurred in 19% of acute and 43% of chronic patients [34]. In an international registry of 137 patients, false lumen thrombosis reached 99.3% in the mid-descending thoracic aorta but only 13.9% in the distal abdominal aorta, and chronic dissection showed more negative remodeling in the descending thoracic aorta than acute dissection (33% versus 17.5%) [37]. Volumetric analysis of 63 acute DeBakey type I dissections showed first-year false lumen thrombosis of 98%, 68%, and 39% in the stented, thoracoabdominal, and abdominal segments; thrombosis correlated inversely with total entry size and the number of branches arising from the false lumen, and the exit number predicted negative remodeling [38]. A meta-analysis of 43 studies and 5068 patients reported a pooled false lumen thrombosis rate of 91% at the stent level and a secondary TEVAR rate of 7%, with pooled dSINE and endoleak rates of 2% and 3% and very high heterogeneity [14]. The gap between pooled estimates and single-center rates likely reflects differences in follow-up duration, imaging intensity, and treatment strategy. The prognostic weight of these findings comes from primary type B dissection rather than from patients treated with FET: partial false lumen thrombosis independently predicted post-discharge mortality after acute type B dissection (relative risk 2.69) [39], and in patients with a patent false lumen the size and location of the entry tear predicted late events [40].

### 4.3. Distal Stent Graft-Induced New Entry

dSINE is a new intimal tear at the distal edge of the stent graft and is a characteristic failure mode of FET in dissection. In 126 patients treated with the Thoraflex device at two centers, dSINE occurred in 13%, the cumulative risk reached 14%, 16%, and 25% at 12, 24, and 36 months, and no predictor was identified [15]. A scoping review of 544 FET procedures reported dSINE in 12.7% of patients, with mean times to diagnosis of 12.6 to 30.6 months after surgery across studies, and the Bologna group added 54 cases among 225 FET procedures [41]. In 52 patients treated with the J Graft FROZENIX, dSINE occurred in 23% and was associated with chronic dissection, a larger residual false lumen area, and cranial migration of the distal edge [42]. Chronic dissection is the highest-risk setting: dSINE developed in 22 of 43 patients (51.1%) over a mean of 5.9 years, a total aortic diameter above 45 mm at the distal stent level was independently associated with dSINE (hazard ratio 4.60, 95% confidence interval 1.01 to 20.85), and 5-year freedom from dSINE was 87.5% versus 20.8% below and above that threshold [16]. Oversizing matters but is sensitive to how it is measured; among three measurement methods, perimeter-based true lumen oversizing discriminated dSINE best in a recent series of 97 patients with seven events, with an exploratory cutoff of 17% [43]. Longitudinal imaging shows that aortic elongation along the outer curvature precedes dSINE, whereas stent migration and the bird-beak angle increase afterward, and dSINE was more frequent after FET than after TEVAR (32% versus 10%) in one comparative cohort [44]. Finite element and computational fluid dynamics models explain this pattern: elastic recoil pushes the distal edge against the greater curvature, oversizing concentrates circumferential wall stress, and undersizing raises wall shear stress, so smaller devices alone may not prevent dSINE [45,46]. Evidence from TEVAR for type B dissection carries the same message. In type B dissection, pooled dSINE incidence after TEVAR is 10.1%, the relative risk in chronic compared with acute disease is 3.12, and excessive distal oversizing roughly doubles the odds of dSINE [47], with incidence rates of 12.9% in chronic and 4.3% in acute repairs [48]. Every secondary TEVAR therefore creates a new distal edge that carries its own dSINE risk.

## 5. Predictors of Incomplete Remodeling

The available predictors fall into five groups (Table 1). The first is device coverage: short stents, a proximally located distal end, and zone 2 anastomoses without a compensatory increase in stent length are associated with more secondary interventions [23,26], whereas longer stents improve thoracic remodeling at the cost of a higher risk of spinal cord ischemia [28,30]. The second is residual entries and false lumen outflow. In the analysis of 186 dissections, patients with distal aortic failure did not have more communications overall, but they had more communications in zones 6 to 8, the paravisceral aorta from the celiac trunk to the renal arteries (*p* = 0.01), and not in the descending thoracic zones 4 and 5 (*p* = 0.11); acute presentation, preoperative maximal descending diameter, and diameter at the diaphragm were independent risk factors [12]. Re-entries in zones 6 to 8 cannot be sealed by a thoracic TEVAR that ends above the celiac trunk. The number of exits and the total entry size predict persistent false lumen perfusion downstream [38]. The third is chronicity. Chronic dissection is associated with more negative descending remodeling [37], more late deterioration [34], and the highest dSINE rates [16,42]. The discordance between the acute dissection signal in one series [12] and the chronic dissection signal in others likely reflects different endpoints: acute cases fail by persistent false lumen perfusion and growth, chronic cases by dSINE and the inability of a stiff flap to reappose.

The fourth group is aortic size and early trajectory. A total aortic diameter above 45 mm at the planned distal stent level was independently associated with dSINE in 43 chronic dissections, although with a wide confidence interval [16]. In 56 patients operated on for chronic dissection, early negative remodeling, defined as any increase of at least 1 mm in the proximal descending diameter between the preoperative and the 6-month CT after FET, was associated with 5-year freedom from distal reintervention of 6.2%, compared with 44.6% after early positive remodeling, and with 5-year dSINE-free survival of 18.2% compared with 65.1% [49]. In that study, patients with early negative remodeling who did not undergo distal intervention had lower 5-year survival than those who did (0% versus 40.5%), but the analysis was unadjusted, excluded patients who died within 6 months, and compared treated with untreated patients without accounting for the time to intervention, so it cannot establish a benefit of intervention; overall 5-year survival did not differ between the remodeling groups (83.6% versus 77.2%) [49]. Aortic geometry also contributes. Oversizing, aortic elongation, and cranial migration of the distal edge precede dSINE [42,43,44], and shape analysis of 93 post-FET aortas linked the angle at the isthmus to later aortic events [50]. The fifth group is heritable thoracic aortic disease. Marfan syndrome was an independent risk factor for in-hospital mortality after FET [13], and dedicated series in Marfan syndrome and hereditary aortopathy show acceptable early results but substantial downstream reintervention [51,52,53]; in one series, 18 of 34 patients with Marfan syndrome required distal reintervention, most often open [52]. TEVAR in connective tissue disease is not a definitive option: in 40 patients, 71.1% of survivors developed aneurysm-related morbidity, most often aneurysmal degeneration [54]. In the largest single-center dissection cohort, however, connective tissue disease was not an independent risk factor for distal aortic failure [12]. Connective tissue disease and a baseline aneurysm above 55 mm were risk factors for aortic events after second-stage endovascular repair in another series [55].

**Table 1:** Anatomic, procedural, and patient-related predictors of incomplete distal remodeling and distal stent graft-induced new entry after the frozen elephant trunk.

Predictor	Key Evidence	Direction of Effect	Implication for Secondary TEVAR
Short stent graft, proximal distal end, zone 2 anastomosis	232 patients: highest secondary intervention rate with zone 2, 100 mm stent ending at Th2 to Th3 [23]; E-vita versus Thoraflex, 0% versus 22% secondary endovascular interventions [26]	Less coverage, more reintervention	Expect a second stage; plan overlap and distal landing at the index operation
Stent graft of 150 mm or more, or distal end at or below Th8	Better thoracic remodeling with 150 mm stents [28]; residual malperfusion 2.7% versus 6.5%, *p* = 0.62 [24]; spinal cord ischemia 11.6% versus 2.5% with coverage of at least 15 cm or to Th8 [30]; distal end below Th9, odds ratio 15.2 [31]	Better thoracic remodeling, higher spinal cord risk	Shorter native landing zone above the celiac trunk; greater segmental artery consumption
Re-entries in zones 6 to 8 (paravisceral); number of exits	More communications in zones 6 to 8 (*p* = 0.01), not in zones 4 and 5 (*p* = 0.11), in patients with distal aortic failure [12]; exits predict negative remodeling [38]	Persistent false lumen perfusion	Not sealed by thoracic TEVAR; consider false lumen occlusion, F/BEVAR, or surveillance
Chronic dissection	Negative descending remodeling 33% versus 17.5% [37]; dSINE 51.1% [16]; dSINE 23% linked to chronicity [42]	Stiff flap, high dSINE risk	Early completion with minimal oversizing; consider adjuncts
Total aortic diameter above 45 mm at distal stent level	Hazard ratio 4.60 (95% CI 1.01 to 20.85) in 43 chronic dissections; 5-year freedom 87.5% versus 20.8% [16]	dSINE	Measure early after FET; imprecise estimate that requires validation
Early negative remodeling at 6 months (chronic dissection)	Freedom from distal reintervention 6.2% versus 44.6%; unadjusted analysis of 56 patients [49]	Distal failure	Use the 6-month CT after FET as a decision point in chronic dissection
Oversizing and geometry	Perimeter-based oversizing of 17% or more, exploratory cutoff with 7 events [43]; elongation precedes dSINE [44]	dSINE	True lumen perimeter-based sizing; plan margin for elongation
Heritable thoracic aortic disease	Marfan syndrome predicted mortality after FET [13]; distal reintervention in 18 of 34 patients with Marfan syndrome [52]; TEVAR not definitive in connective tissue disease [54]	Aneurysmal degeneration	Prefer open distal repair; TEVAR as bridge or graft-to-graft

CI, confidence interval; CT, computed tomography; dSINE, distal stent graft-induced new entry; F/BEVAR, fenestrated or branched endovascular aortic repair; FET, frozen elephant trunk; Th, thoracic vertebra; TEVAR, thoracic endovascular aortic repair.

## 6. Indications for Secondary TEVAR

Reinterventions after FET fall into three categories: intended completion, anticipated reintervention triggered by disease progression, and unexpected reintervention for complications [9,11]. Intended completion applies when the FET cannot, by design, treat the full extent of disease. Typical examples are degenerative aneurysms extending beyond the stent graft, chronic dissections with established descending aneurysm, and residual dissections with known large entries in the mid or distal descending aorta. In these patients, the second stage is part of the operative plan, and the main point of interest is the appropriate timing. For extensive aneurysm, the interval between stages carries its own risk: after conventional elephant trunk repair, 4 of 111 patients died while awaiting the second stage, 3 of them from rupture [56]. Anticipated reintervention is triggered by surveillance findings: diameter growth, persistent false lumen perfusion with negative remodeling, or an asymptomatic dSINE. Guideline thresholds for chronic dissection, generally a maximal diameter of approximately 55 mm or rapid growth, apply [7,20,57], but post-FET series have also intervened for growth of more than 5 mm within 6 months or for dSINE, which accounted for 47% and 18% of 66 downstream TEVAR procedures in one series [58]. In one series of 37 extensions among 232 FET patients, the maximal aortic diameter increased from 40.8 mm after FET to 45.1 mm before TEVAR, and only 37.8% of patients had complete false lumen or aneurysm occlusion before extension [59].

Unexpected reinterventions include malperfusion that persists after the index operation, rupture, symptomatic dSINE, and endoleak. A late failure mode specific to the FET and TEVAR interface also deserves attention: in 30 patients who received TEVAR extensions covering most of a 10 cm FET module with 2 to 4 mm oversizing, three (10%) developed type III endoleak from disconnection between the two devices 34 to 45 months later; no risk factor was identified, and the authors recommended annual CT angiography without time limit [60]. Distal false lumen perfusion from paravisceral, abdominal, or iliac re-entries can persist after complete thoracic coverage and may require false lumen occlusion or branched extension, discussed below. The indication for secondary TEVAR is therefore rarely a single threshold. It integrates the extent of residual disease, the location of re-entries, the status of the false lumen, the trajectory on serial imaging, the presence of complications, and the patient’s clinical condition.

## 7. Timing of Secondary TEVAR: Planned Versus Reactive

### 7.1. The Biological Window: Evidence from Primary Type B Dissection

The case for early completion rests on the time-dependent plasticity of the dissection flap, and almost all data supporting it come from primary type B dissection, in which time is measured from symptom onset. In acute uncomplicated type B dissection, the randomized ADSORB trial showed that TEVAR added to best medical treatment promoted false lumen thrombosis and remodeling at 1 year [61]. INSTEAD-XL enrolled 140 patients with stable type B dissection 2 to 52 weeks after onset; TEVAR reduced aorta-specific mortality at 5 years (6.9% versus 19.3%) and disease progression (27.0% versus 46.1%), although all-cause mortality did not differ significantly, and these benefits were associated with stent graft-induced false lumen thrombosis in 90.6% of cases [62]. In the VIRTUE registry, subacute dissections remodeled as well as acute ones, whereas chronic dissections showed less aortic plasticity and more reinterventions (39%) [63]. In 110 patients treated at least 2 weeks after onset, complete thoracic remodeling at 1 year was achieved in 88.4% when TEVAR was performed within 3 months of onset, 57.1% between 3 and 12 months, and 18.5% after 12 months, and an interval longer than 3 months was the strongest predictor of failure (odds ratio 9.75) [35]. Flap motility, measured as true lumen strain, was greater in acute and subacute than in chronic primary or residual type B dissections treated with TEVAR or FET, and true lumen expansion was 9, 5, and 2 mm, respectively [64]. A network meta-analysis of 17 studies found that subacute TEVAR had lower short-term mortality, fewer strokes, and lower long-term all-cause mortality than acute TEVAR [65], and a reconstructed time-to-event meta-analysis of 4793 patients also favored the subacute phase [66].

These populations are not equivalent. After FET, the primary entry and the arch have been treated, the proximal descending aorta is stented, the remaining false lumen flow depends on distal re-entries and branch outflow, and a large proportion of patients have chronic or residual dissection. The type B data therefore support the biological principle that a young flap reapposes better than an old one, but they do not define a window for residual dissection after FET. Direct evidence is limited. In the ERADICARE pilot trial, 32 patients with a re-entry in Ishimaru zones 3 or 4 and a descending diameter above 40 mm or a false lumen diameter above 20 mm after surgery for acute type I dissection were randomized to TEVAR 1 to 3 months after surgery or to conservative management; TEVAR improved 1-year remodeling and reduced dissection extension (0% versus 29.4%) and adverse aortic events (20.0% versus 58.8%) [18]. The trial was small and was not restricted to patients treated with FET. A multicenter randomized trial of early endovascular repair for a dominant false lumen with residual thoracic tears after total arch replacement with FET, with 5-year all-cause mortality as the primary endpoint, is ongoing [19].

### 7.2. Planned Second-Stage TEVAR

A planned second stage aims to seal residual entries in the descending thoracic aorta while the flap is still compliant, before growth and before dSINE, and to separate the spinal cord insult of the index operation from that of the completion. Most published series mix planned, anticipated, and urgent procedures, and they report intervals from the index FET rather than from dissection onset. In these series, early mortality after endovascular extension ranged from 0% to 6.6% and permanent paraplegia from 0% to 3.3% [58,60,67]. Among 66 downstream TEVAR procedures performed a median of 7 months after zone 2, 100 mm FET, 26% were planned completions, 47% were performed for diameter progression, and 18% for dSINE; cerebrospinal fluid drainage was used in 92%, one patient (2%) had temporary spinal cord injury, and there was no in-hospital death, stroke, or permanent spinal cord injury, although 15 patients required a further aortic reintervention after a median of 12 months [58]. In 10 patients treated a mean of 136 days after FET, spinal cord preconditioning was used in 70%, no paraplegia occurred, and complete false lumen thrombosis was achieved in all type B dissections [67]. A series of 34 s-stage procedures after FET for type A dissection, 26 of them using the STABILISE technique, reported no perioperative death or major complication and significant distal remodeling in all segments [55]. In a national aortovascular center, 32 patients underwent a two-stage approach with median intervals of 140 to 240 days after the first stage and no in-hospital or 90-day mortality; spinal cord ischemia was recorded in 9 or 10 patients (the abstract and tables differ) in this mixed endovascular and open second-stage cohort, a reminder that staging reduces but does not eliminate the risk [68]. Other centers report 29 distal extensions without 30-day death and 36-month freedom from reintervention of 72% and 64% in aneurysm and dissection groups [69].

A planned approach does not mean immediate. In a series comparing early extension within 30 days of FET (mean 4.8 days) with late extension (mean 18.4 months after FET), the early group had longer intensive care stays (10.7 versus 0.1 days) and hospitalizations (22.4 versus 8.1 days), 50% respiratory failure, and 28.6% dialysis-dependent kidney failure, whereas post-extension remodeling was similar in the two groups, and the authors recommended delaying extension until recovery [59]. These results reflect that very early extensions are usually performed during the index admission for malperfusion, rupture, or other complications, not that elective early completion is harmful. Early imaging may help with scheduling, but the only data come from chronic dissection, in which the direction of change between the preoperative and the 6-month CT after FET was associated with later distal failure in an unadjusted analysis [49]. No study has tested a decision scan after FET for acute dissection. Our suggestion of a decision CT at 6 to 8 weeks after FET, which leaves time for completion before day 90 from onset, combines the type B timing data with the morbidity of very early extension; it is an interpretation, not a tested strategy.

### 7.3. Surveillance-Based Reactive TEVAR

The surveillance approach is supported by the fact that most patients never need distal treatment. False lumen thrombosis at the stent level is achieved in approximately 91% of patients [14], and freedom from distal reintervention exceeds 80% at 10 years in pooled data [10]. Reactive TEVAR avoids additional spinal cord risk, contrast exposure, cost, and a second access procedure in patients who would remodel spontaneously, and treatment of dSINE with a short distal extension is technically simple with low morbidity [9]. The weaknesses are equally clear. dSINE is usually asymptomatic [41], surveillance must be lifelong, and, by extrapolation from type B dissection, a flap treated late is stiffer and less likely to reappose [35,64]. Delay also increases the size of the aorta that must eventually be treated. In 106 patients undergoing open thoracoabdominal repair after FET, a diameter of 60 mm or more at the distal end of the FET identified patients with worse early outcomes, including early death [70]. After redo FET for residual type A dissection, 39% of 110 survivors required at least one reintervention within 48 months, most of them endovascular [71]. A reactive strategy is reasonable when reliable lifelong surveillance can be ensured and early imaging shows favorable remodeling.

### 7.4. Published Outcomes and Synthesis

Table 2 summarizes published series reporting outcomes of secondary endovascular treatment after FET, and Table 3 contrasts the two strategies. Across series of mixed elective and nonelective procedures, secondary TEVAR is associated with low early mortality and few permanent spinal cord deficits in experienced centers, whereas the burden of later reintervention remains substantial, ranging from roughly one in five to one in three patients within 2 to 3 years [58,60,69]. The evidence does not support a universal timing rule. It does support three cautious conclusions. First, extensions performed during the index admission for complications carry high morbidity and should not be used to judge elective completion [59]. Second, in the only randomized study, TEVAR 1 to 3 months after type I repair improved 1-year remodeling in selected high-risk patients [18]. Third, patients at risk can often be identified from preoperative anatomy and early imaging, although the predictive thresholds come from small series [12,16,49]. Timing should therefore be individualized to the patient’s anatomy, remodeling trajectory, chronicity, and clinical condition rather than set by a fixed calendar.

## 8. Technical Considerations

### 8.1. Proximal Seal Within the Hybrid Prosthesis

The FET stent graft offers a circumferential, nondissected, prosthetic landing zone of known diameter, which makes the proximal seal of the completion TEVAR more predictable than in native aorta [9,73]. Sizing follows the internal diameter of the FET rather than native aortic measurements, and oversizing of 2 to 4 mm relative to the FET has been used successfully [60]; larger oversizing inside the stented segment may cause infolding. The overlap should be generous and should cover the distal end of the FET completely, both to exclude the dSINE-prone edge and to reduce the risk of later disconnection, which occurred in 3 of 30 patients in one series despite coverage of most of the 10 cm FET stent graft [60]. Guidewire access must be confirmed in the true lumen along its entire course, particularly when fenestrations or dSINE create alternative channels, and intravascular ultrasound is useful for this purpose. The cranial migration of the FET distal edge and the progressive elongation of the descending aorta described after dSINE argue for planning overlap with margin for future movement rather than for a minimal connection [42,44].

### 8.2. Distal Landing in a Dissected Aorta

The distal landing zone of a completion TEVAR usually lies in the dissected aorta. After TEVAR for acute descending dissection, dissected distal landing zones enlarged by 7 mm in the true lumen and 4 mm in total diameter within 1 year, and 10 distal stent graft-induced new entries developed among 101 patients [74]. The distal end should land in a straight segment, sized to the true lumen with minimal oversizing, using tapered devices when the true lumen is small, because excessive distal oversizing and chronicity are the main determinants of dSINE after TEVAR [47,48]. Coverage close to the celiac trunk carries a spinal cord cost: after TEVAR, patients with spinal cord ischemia had a shorter uncovered segment above the celiac artery (17 versus 63 mm), and patients with permanent deficits had longer coverage (261 versus 196 mm) [75]. When the dissection extends to the visceral segment, the goal of completion is usually to seal the dominant thoracic re-entries, ending above the celiac trunk, with acceptance of residual abdominal false lumen flow, followed by surveillance or distal adjuncts.

### 8.3. Spinal Cord Protection

Spinal cord ischemia is the main risk of completion. A long FET combined with one or two thoracic stent grafts easily exceeds 20 cm of covered aorta and can extend from the arch to the thoracoabdominal junction. The European Registry of Endovascular Aortic Repair Complications identified simultaneous closure of at least two of the four vascular territories supplying the spinal cord (left subclavian, intercostal, lumbar, and hypogastric arteries) and prolonged hypotension as the mechanisms of symptomatic spinal cord ischemia after TEVAR [76]. In acute DeBakey type I dissection treated with FET, segmental arteries between the ninth thoracic and third lumbar levels that depended entirely on the false lumen were present in 24% of patients and were associated with a higher spinal cord injury rate (34.3% versus 2.7%; odds ratio 13.1) [77]. This finding has direct implications for completion TEVAR, because thrombosis of the false lumen sacrifices segmental arteries that originate from it.

Staging is itself a spinal cord protection strategy. In open thoracoabdominal repair, permanent spinal cord injury occurred in 15% of patients after single-stage repair and in none after two-stage repair, despite more segmental arteries sacrificed in the staged group [78]; separating the arch and descending repairs is expected to give the collateral network time to adapt in the same way, and minimally invasive staged segmental artery coil embolization extends this principle by occluding up to seven segmental arteries per session at least 5 days before the definitive repair; its efficacy is being tested in the randomized PAPAartis trial [72,79]. Preservation or revascularization of the left subclavian artery at the index FET keeps one territory open. In 672 matched pairs undergoing TEVAR for type B dissection, preoperative cerebrospinal fluid drainage was associated with less permanent spinal cord ischemia (1.3% versus 3.4%) but not less transient ischemia [80], and drainage was used in 92% of downstream TEVAR procedures in one post-FET series without permanent spinal cord injury [58]. Guidelines support selective drainage for patients at high risk, maintenance of adequate perfusion pressure, and avoidance of hypotension [7].

### 8.4. Distal Bare Metal Stents and False Lumen Occlusion

When the dissection extends into the abdominal aorta, several adjuncts can promote true lumen expansion or false lumen exclusion beyond the covered segment. The provisional extension to induce complete attachment (PETTICOAT) concept added bare metal stents distal to the stent graft to expand the true lumen [81], and the composite device design was evaluated prospectively in the STABLE studies, with 5-year results in acute complicated type B dissection [82]. The stent-assisted balloon-induced intimal disruption and relamination (STABILISE) technique goes further, deliberately rupturing the flap within the bare stent to create a single lumen [83], and it was used in most second-stage procedures in one post-FET series, with greater distal remodeling in treated patients [55]. Bare metal stents do not seal re-entries, their effect depends on flap compliance, and they can make later open thoracoabdominal surgery technically difficult because they adhere strongly to the intima [84]. For persistent false lumen backflow in chronic dissection, the candy-plug and knickerbocker techniques occlude the false lumen at the level of the distal thoracic aorta [85,86], and a systematic review of candy-plug use in type B and residual type A dissection supports its role in selected patients [87].

### 8.5. Beyond TEVAR: Third-Stage Repair

Some patients require a third stage. After TEVAR extension, 5 of 30 patients in one series and 9 of 29 in another progressed to fenestrated or branched thoracoabdominal endovascular repair [60,69]. Open thoracoabdominal replacement remains an option for young patients and for those with heritable aortopathy, and the prior TEVAR extension moves the proximal anastomosis distally, which simplifies the operation [9]. The size of the aorta at the time of the third stage matters: a diameter of 60 mm or more at the distal end of the FET was associated with early death after open thoracoabdominal repair [70]. The three-stage pathway argues against allowing the post-FET aorta to enlarge while awaiting symptoms.

## 9. Overlap with Branched Arch Endografts

Total endovascular arch repair has matured into an alternative for selected patients who would otherwise require FET followed by TEVAR. In a global feasibility study of 39 patients treated with three-vessel inner branch stent grafts, technical success was 100%, 30-day mortality 5%, and secondary interventions were needed in 31% [88]. In the chronic dissection arm of the NEXUS investigational device exemption study, 54 high-risk patients, 70% with previous sternotomy, underwent zone 0 repair with 98.2% technical success, 9.3% 30-day mortality, and no spinal cord ischemia [89]. The most direct comparison addresses residual dissection after ascending repair for type A dissection. After propensity matching of 114 patients, branched endovascular arch repair and open arch repair had similar 30-day mortality (5.3% versus 3.5%) and stroke (3.5% versus 5.3%), whereas branched repair reduced prolonged ventilation, acute kidney injury, and hospital stay (10 versus 21 days) [90]. Redo FET remains an option in this population, but it carries a reintervention risk of 55.4% at 4 years [71,91].

These developments create overlap rather than replacement. For a patient with residual arch and descending dissection after ascending repair, the choice now lies between redo FET followed by completion TEVAR and a branched endovascular arch combined with thoracic extension in one or two sessions. The endovascular route avoids circulatory arrest and resternotomy but does not change the distal problem: the same coverage length, the same spinal cord territories, and the same dSINE biology apply at the new distal edge. In our view, single-session total endovascular repair from zone 0 to the thoracoabdominal junction concentrates spinal cord risk in one procedure and should generally be staged. In the acute type A setting, noncovered hybrid stents such as the Ascyrus Medical Dissection Stent represent a different compromise; pooled analyses favor FET for false lumen thrombosis, neurological complications, and early mortality, and long-term downstream data for noncovered stents are limited [92,93,94].

## 10. Surveillance and Emerging Tools

A patient who has undergone FET is never cured of aortic disease. Surveillance after FET should therefore be structured and lifelong, within an aortic center that can offer secondary endovascular and open treatment [7,9]. We suggest CT angiography before discharge, at 6 to 8 weeks after FET for acute dissection so that a subacute completion remains possible, at 6 and 12 months, and yearly thereafter; this schedule reflects our interpretation rather than tested evidence. Annual imaging should continue after completion TEVAR because disconnection and new distal entries can occur years later [60]. Diameter alone is an insensitive marker of risk, and several tools may refine phenotyping. In type B dissection, false lumen ejection fraction measured with 4D flow magnetic resonance imaging predicted aortic growth [95]. Deep learning segmentation of residual aortic dissection on CT showed that local false lumen volume predicted adverse remodeling better than maximal diameter (area under the curve 0.83 versus 0.73) in internal validation and retained its advantage in an external cohort [96]. After FET specifically, an unsupervised variational autoencoder produced a continuous false lumen thrombus score that tracked individual remodeling trajectories on serial scans [97]. None of these tools has yet been validated as a trigger for completion TEVAR, but together they point toward risk stratification based on false lumen hemodynamics and volumes rather than on diameter thresholds alone.

## 11. A Proposed Phenotype-Driven Approach

**Figure 2:**
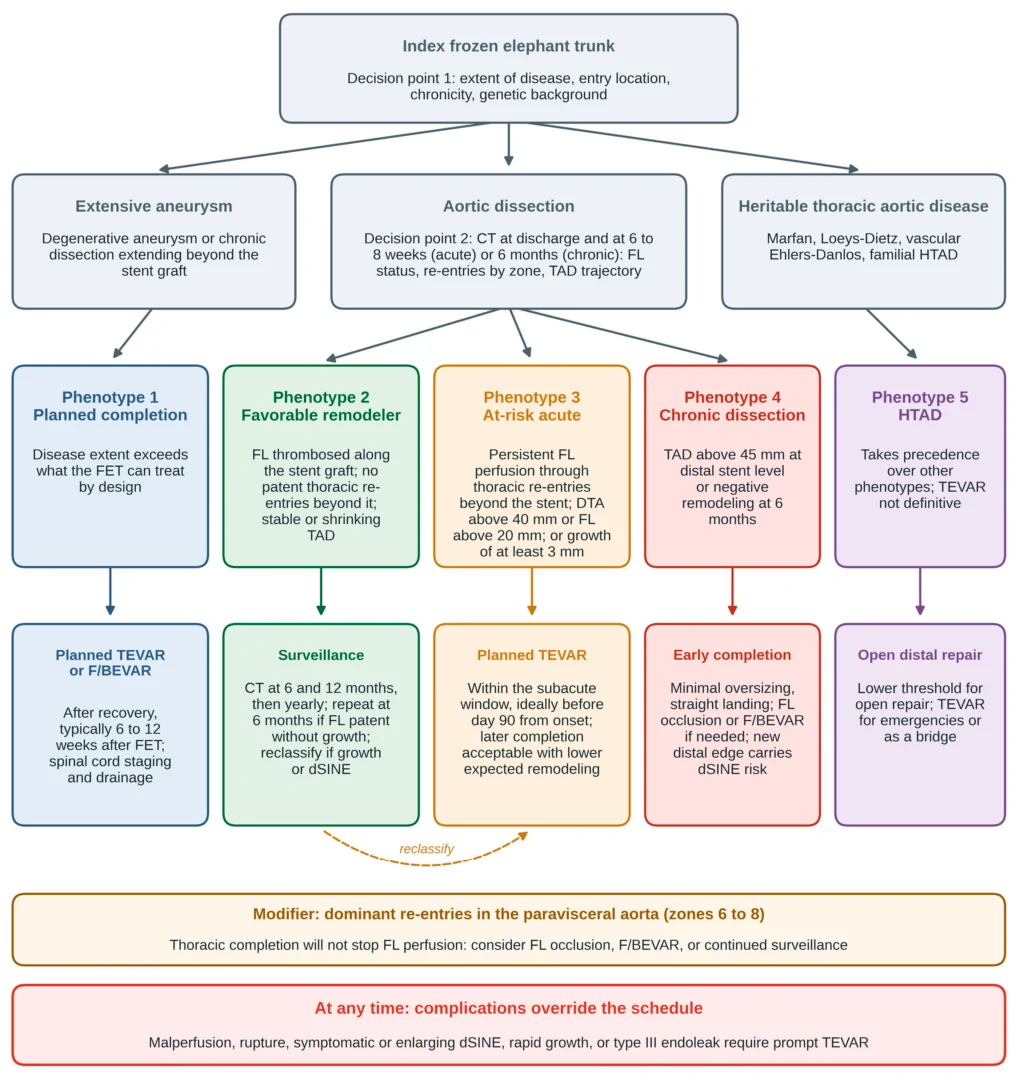
Proposed phenotype-driven approach for secondary thoracic endovascular aortic repair (TEVAR) after the frozen elephant trunk (FET). Decisions are made at the index operation and on early imaging: at discharge and 6 to 8 weeks after FET for acute dissection, and 6 months after FET for chronic dissection. The approach has not been validated; thresholds derive from small observational series and one pilot randomized trial and are intended as hypotheses for prospective testing. Phenotypes may overlap, and decisions should be individualized. CT, computed tomography; dSINE, distal stent graft-induced new entry; DTA, descending thoracic aorta; F/BEVAR, fenestrated or branched endovascular aortic repair; FL, false lumen; HTAD, heritable thoracic aortic disease; TAD, total aortic diameter.

We propose, as a hypothesis for prospective testing rather than a validated algorithm, that the decision between planned and reactive distal extension be made at two points: at the index operation, when the extent of disease, the location of re-entries, chronicity, and genetic background are known, and on early imaging, at 6 to 8 weeks after FET for acute dissection and at 6 months after FET for chronic dissection (Figure 2). Table 4 shows which elements of the approach rest on direct evidence after FET, which are extrapolated from other settings, and which reflect our interpretation. Complications override the schedule in every phenotype, and heritable thoracic aortic disease takes precedence over the other phenotypes. Phenotype 1, planned completion, includes degenerative aneurysms extending beyond the stent graft and chronic dissections with a descending aorta already near intervention size; completion by TEVAR, or by fenestrated or branched repair when the disease reaches the visceral segment, should follow recovery, typically 6 to 12 weeks after FET, because deaths while awaiting a second stage are mostly due to rupture [56]. Phenotype 2, the favorable remodeler, includes acute dissections with false lumen thrombosis along the stent graft, no patent re-entries in the descending thoracic aorta beyond the stent, and a stable or decreasing total aortic diameter; surveillance is appropriate, and a patent false lumen without growth warrants repeat imaging at 6 months after FET. Phenotype 3, the at-risk acute dissection, includes persistent false lumen perfusion through re-entries in the descending thoracic aorta beyond the stent with a descending diameter above 40 mm or a false lumen diameter above 20 mm, criteria adapted from the only randomized study [18], or growth of at least 3 mm on early imaging, a pragmatic threshold that we chose to exceed typical measurement variability. Completion should be planned within the subacute window, ideally before day 90 from onset; later completion remains reasonable, with lower expected remodeling by extrapolation from type B dissection [35,64]. When the dominant re-entries lie in the paravisceral aorta (zones 6 to 8), thoracic completion will not stop false lumen perfusion, and false lumen occlusion, fenestrated or branched repair, or continued surveillance should be considered [12,87].

**Figure 3:**
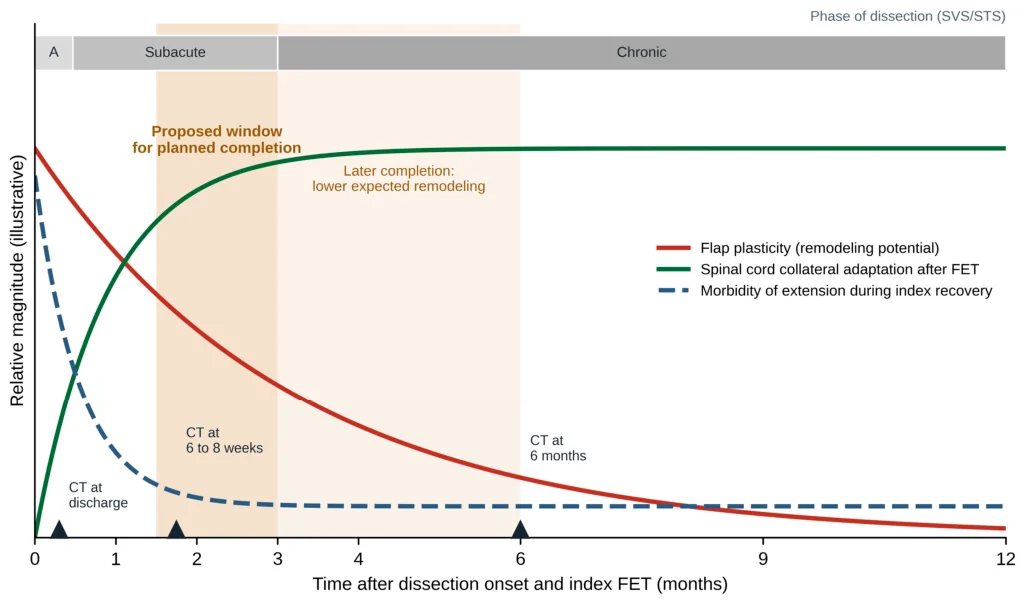
Conceptual timeline for completion thoracic endovascular aortic repair (TEVAR) after the frozen elephant trunk (FET) for acute dissection. Time is measured from dissection onset; for acute dissection treated with FET within days of onset, it approximates the time after FET. Flap plasticity declines over the first months, the spinal cord collateral network adapts after the index operation, and the risk of an early reintervention during the index recovery is high. The darker shaded region marks the proposed elective window for planned completion, derived by extrapolation from type B dissection; the lighter region indicates later completion with lower expected remodeling. Phase boundaries follow the Society for Vascular Surgery and Society of Thoracic Surgeons reporting standards (A, acute) [21]. Curves are illustrative and are not derived from pooled data.

Phenotype 4, chronic dissection, carries the highest dSINE risk and the least flap plasticity. A total aortic diameter above 45 mm at the distal stent level or negative remodeling on the 6-month CT after FET supports early completion with minimal oversizing and a straight distal landing zone, supplemented by false lumen occlusion techniques or planned fenestrated, branched, or open repair when thoracic coverage alone cannot achieve exclusion [16,49,86]; the new distal edge carries its own dSINE risk, 12.9% after TEVAR for chronic dissection [48]. Phenotype 5, heritable thoracic aortic disease, requires a lower threshold for open distal repair; TEVAR should serve mainly in emergencies or as a bridge to open thoracoabdominal replacement [7,54]. Individual patients may show features of more than one phenotype, for example an acute dissection with both thoracic and paravisceral re-entries, or a patient whose trajectory changes between scans. The phenotypes are therefore a structure for discussion within the aortic team, not mutually exclusive categories, and the final decision should integrate aortic anatomy, disease progression, comorbidity, frailty, and patient preference. The thresholds derive from small observational series and one pilot trial and require prospective validation. Figure 3 illustrates the conceptual balance between flap plasticity, spinal cord collateral adaptation, and procedural risk that underlies the proposed window.

## 12. Knowledge Gaps and Future Directions

The evidence on secondary TEVAR after FET is largely retrospective, often from overlapping single-center cohorts, device-specific, and inconsistent in its definitions of remodeling, reintervention, and timing. Intended completions, anticipated reinterventions, and emergency extensions are frequently pooled, which obscures the effect of timing. Several steps would change practice. First, reporting should adopt the ARCH Academic Research Consortium endpoints and the SVS and STS chronicity windows, and it should specify the interval from dissection onset, not only from the index operation [21,36]. Second, multicenter registries should capture the discharge and early imaging phenotype, including re-entry location by zone, false lumen-dependent branches, and total aortic diameter at the distal stent level, so that predictive models can be built and externally validated. Third, the timing data from primary type B dissection need direct confirmation in residual dissection after FET. The ERADICARE pilot and the ongoing multicenter trial after FET [18,19] should be followed by adequately powered trials comparing planned completion within the subacute window with imaging-triggered TEVAR; their primary endpoint should combine aortic-related death, rupture, unplanned reintervention, and spinal cord injury. Fourth, quality of life, sex-specific outcomes, cumulative radiation, and cost should be reported, as the first studies of sex differences in staged arch repair have begun to do [68]. Finally, automated volumetric and hemodynamic imaging should be tested as triggers for intervention rather than as descriptive tools [96,97].

## 13. Conclusions

Secondary TEVAR after FET is common and, in experienced centers, associated with low early mortality and few permanent deficits, although published series mix elective and urgent procedures. Distal failure rates depend on strategy, device, case mix, and follow-up; dSINE is a characteristic failure mode, particularly in chronic dissection, and paravisceral re-entries are beyond the reach of thoracic completion. Short stent coverage, larger descending and distal stent-level diameters, chronicity, early negative remodeling, and heritable aortopathy identify patients at risk. A universal timing rule is not supported by current evidence, and most data on the time-dependent loss of flap plasticity come from primary type B dissection rather than from patients treated with FET. We propose a phenotype-driven approach, with planned completion within the subacute window from dissection onset for persistent thoracic false lumen perfusion, surveillance for favorable remodeling, careful attention to sizing, overlap, and spinal cord protection, and lifelong imaging, as a hypothesis for prospective testing. Timing should be individualized to aortic anatomy, disease progression, and the patient’s clinical condition. Above all, FET does not cure the aorta: every patient remains subject to lifelong surveillance in an aortic center that can offer secondary endovascular and open treatment.

## Figures and Tables

**Table 2:** Selected series reporting secondary endovascular treatment after the frozen elephant trunk.

Study	Population	Interval from FET	Early Outcomes	Later Events
Kreibich 2020 [11]	35 of 107 FET patients reintervened; 24 endovascular	Intended, anticipated, and unexpected	In-hospital mortality 14% after any reintervention (24 endovascular, 7 open, 4 hybrid); no stroke or permanent SCI	Cumulative reintervention risk 31%, 49%, 64% at 12, 24, 36 months
Haensig 2020 [67]	10 s-stage endovascular repairs (TEVAR or branched)	Mean 136 days	No paraplegia; 70% spinal cord preconditioning	Complete false lumen thrombosis in type B dissections
Leone 2020 [13]	437 FET, two European institutes	Not specified	Not reported separately	61 endovascular extensions (16.3% of hospital survivors); 25 open (6.7%)
Kreibich 2022 [58]	66 downstream TEVAR after zone 2, 100 mm FET; 26% planned	Median 7 months	No in-hospital death, stroke, or permanent SCI; 1 temporary SCI	15 further reinterventions after median 12 months
Hostalrich 2025 [60]	30 of 159 FET patients	Elective and emergency (6 emergencies)	Mortality 6.6%; paraplegia 3.3%	F/BEVAR extension in 5 of 30; disconnection in 3 of 30
Fortin 2024 [55]	34 s-stage procedures after FET for type A dissection (26 STABILISE)	Staged	No perioperative death or major complication	Significant remodeling in all segments
Bruno 2025 [69]	29 TEVAR after FET (12 aneurysm, 17 dissection)	Staged	No 30-day death or early reintervention	36-month freedom from reintervention 72% and 64%
Wenkel 2025 [59]	37 of 232 FET patients	Early mean 4.8 days versus late mean 18.4 months	Early: ICU 10.7 days, dialysis 28.6%, one death; late: ICU 0.1 days	Only 37.8% had complete occlusion before extension
Jhala 2026 [68]	32 two-stage patients (18 endovascular, 14 open second stage)	Median 140 to 240 days	No in-hospital or 90-day mortality	Spinal cord ischemia in 9 or 10 patients; 5-year survival 78% to 87%
Kletzer 2026 [71]	110 survivors of redo FET for residual type A dissection	Within 48 months	30-day mortality after reintervention 6%; no SCI or disabling stroke reported	Reintervention risk 31.0% at 2 years and 55.4% at 4 years

Intervals are measured from the index FET. Series mix planned, anticipated, and urgent procedures, unless stated otherwise. The two Kreibich series derive from overlapping Freiburg cohorts. Years refer to print publication. F/BEVAR, fenestrated or branched endovascular aortic repair; FET, frozen elephant trunk; ICU, intensive care unit; SCI, spinal cord injury; STABILISE, stent-assisted balloon-induced intimal disruption and relamination; TEVAR, thoracic endovascular aortic repair.

**Table 3:** Planned second-stage versus surveillance-based reactive TEVAR after the frozen elephant trunk.

Domain	Planned Second-Stage TEVAR	Surveillance-Based Reactive TEVAR
Typical timing	Residual acute dissection: after recovery from FET and, ideally, before day 90 from onset, with a decision CT at 6 to 8 weeks after FET; aneurysm: 6 to 12 weeks after FET; chronic dissection: after the 6-month CT [21,35,49]	At detection of growth, dSINE, persistent false lumen perfusion, or complications
Best candidates	Aneurysm beyond the stent graft; re-entries in the descending thoracic aorta beyond the stent graft with a descending diameter above 40 mm or a false lumen above 20 mm [18]; chronic dissection with an enlarged distal aorta or early negative remodeling [16,49]	Acute dissection with false lumen thrombosis along the stent graft and a stable or shrinking diameter on early imaging
Remodeling potential	Treats a compliant flap; complete thoracic remodeling 88.4% when TEVAR within 3 months of onset (type B dissection data) [35]	Treats an older, stiffer flap; complete thoracic remodeling 18.5% when TEVAR more than 12 months after onset (type B dissection data) [35]
Spinal cord	Allows planned staging, drainage, and segmental artery preconditioning [58,72]	Avoids additional coverage in patients who would remodel; emergency extensions offer less opportunity for preparation
Main risks	Overtreatment of patients who would remodel; new distal edge with its own dSINE risk [47]	Silent dSINE, rupture, loss to follow-up, and a larger aorta at eventual repair [70]
Evidence	Retrospective series [55,58,67] and one pilot randomized trial after type I repair [18]	Pooled 10-year freedom from distal reintervention 81.5% [10]; no comparative trials after FET

Intervals for type B dissection are measured from onset; intervals after FET are measured from the index operation. CT, computed tomography; dSINE, distal stent graft-induced new entry; FET, frozen elephant trunk; TEVAR, thoracic endovascular aortic repair.

**Table 4:** Source of support for the main elements of the proposed approach: direct evidence after the frozen elephant trunk, evidence extrapolated from other settings, and authors’ interpretation.

Element of the Approach	Direct Evidence After FET	Evidence Extrapolated from Other Settings	Nature of Support
Avoid elective completion during the index admission	Early (mean 4.8 days) versus late extension: longer ICU stay, respiratory failure, dialysis; similar remodeling [59]	None	One retrospective series; confounded by urgent indications
Complete residual acute dissection before day 90 from onset	Pilot randomized trial of TEVAR 1 to 3 months after type I repair, 32 patients, not restricted to FET [18]	Type B dissection: complete thoracic remodeling 88.4% within 3 months versus 18.5% after 12 months [35]; greater flap motility in acute and subacute phases [64]; subacute remodeling similar to acute [63]	Small trial plus extrapolation; window not validated after FET
Decision CT at 6 to 8 weeks after FET (acute dissection)	None	Derived from the type B window and the morbidity of very early extension [35,59]	Authors’ interpretation
Phenotype 3 criteria (DTA above 40 mm or FL above 20 mm with thoracic re-entries)	Inclusion criteria of the pilot trial [18]	None	Adapted trial criteria; not validated as triggers
6-month CT after FET as a decision point in chronic dissection	Early negative remodeling and later distal failure, 56 patients, unadjusted [49]	None	One two-center series; survivor and selection bias
TAD above 45 mm at the distal stent level (chronic dissection)	Hazard ratio 4.60 (95% CI 1.01 to 20.85), 43 patients [16]	None	Single series; imprecise estimate
Paravisceral re-entries are not addressed by thoracic completion	Distal failure associated with communications in zones 6 to 8, not zones 4 and 5 [12]	Anatomy of thoracic coverage	Direct observational evidence
Complete extensive aneurysm 6 to 12 weeks after FET	None specific	Conventional elephant trunk: 4 of 111 interstage deaths, 3 from rupture [56]	Extrapolation and authors’ interpretation
Staging and cerebrospinal fluid drainage for spinal cord protection	No permanent SCI with drainage in 92% of 66 procedures [58]	Open repair: permanent SCI 0% after staged versus 15% after single-stage repair [78]; TEVAR for type B dissection: permanent SCI 1.3% versus 3.4% with drainage [80]	Observational; mostly extrapolated
Lower threshold for open distal repair in HTAD	Distal reintervention in 18 of 34 patients with Marfan syndrome [52]	TEVAR not definitive in connective tissue disease [54]	Observational; conflicting signal in one cohort [12]
Lifelong annual imaging after completion	Disconnection 34 to 45 months after extension [60]	None	Authors’ interpretation

CI, confidence interval; CT, computed tomography; DTA, descending thoracic aorta; FET, frozen elephant trunk; FL, false lumen; HTAD, heritable thoracic aortic disease; ICU, intensive care unit; SCI, spinal cord ischemia; TAD, total aortic diameter; TEVAR, thoracic endovascular aortic repair.

## Data Availability

No new data were created or analyzed in this study. Data sharing is not applicable to this article.

## Supplementary Materials

The following supporting information can be downloaded at https://www.mdpi.com/article/10.3390/jcm15218585/s1. Table S1: PubMed search strategy.

## Abbreviations

The following abbreviations are used in this manuscript:

AMDS	Ascyrus Medical Dissection Stent
CI	Confidence interval
CT	Computed tomography
dSINE	Distal stent graft-induced new entry
DTA	Descending thoracic aorta
EACTS	European Association for Cardio-Thoracic Surgery
ESVS	European Society for Vascular Surgery
F/BEVAR	Fenestrated or branched endovascular aortic repair
FET	Frozen elephant trunk
FL	False lumen
HTAD	Heritable thoracic aortic disease
ICU	Intensive care unit
PETTICOAT	Provisional extension to induce complete attachment
SCI	Spinal cord injury
STABILISE	Stent-assisted balloon-induced intimal disruption and relamination in aortic dissection repair
STS	Society of Thoracic Surgeons
SVS	Society for Vascular Surgery
TAD	Total aortic diameter
TEVAR	Thoracic endovascular aortic repair
Th	Thoracic vertebra
