# Supplementary material for: Secondary TEVAR After Frozen Elephant Trunk: Indications and Timing
Source: J Clin Med. 2026 Nov 4;15(21):8585. doi: 10.3390/jcm15218585 (PMC13764441; doi:10.3390/jcm15218585)
Supplement: Supplementary file 1 [file jcm-15-08585-s001.zip › jcm-4632390-supplementary.pdf]

## SUPPLEMENTARY MATERIAL

### Secondary Thoracic Endovascular Aortic Repair After Frozen Elephant Trunk: Indications and Timing

### Table S1: PubMed search strategy

PubMed (National Library of Medicine) was searched on 23 and 24 September 2026 without date or language restrictions. Searches were run as free-text strings; records were screened by title and abstract, and reference lists of included reviews and guidelines were screened by hand. Records retrieved by more than one search were counted once at screening.

| No. | Search string | Records |
|---|---|---|
| 1 | frozen elephant trunk AND (TEVAR OR endovascular) AND (secondary OR reintervention OR distal extension OR completion) | 267 |
| 2 | frozen elephant trunk AND (dSINE OR "stent graft-induced new entry" OR "stent-graft induced new entry") | 77 |
| 3 | frozen elephant trunk AND (remodeling OR remodelling OR "false lumen") | 305 |
| 4 | frozen elephant trunk AND (timing OR staged OR "second stage" OR "two-stage") | 154 |
| 5 | frozen elephant trunk AND ("spinal cord" OR paraplegia) AND (stent length OR coverage) | 48 |
| 6 | frozen elephant trunk AND (meta-analysis OR "systematic review") | 34 |
| 7 | (PETTICOAT OR STABILISE OR STABLE) AND aortic dissection AND (bare stent OR bare-metal stent) | 74 |
| 8 | ("candy plug" OR "candy-plug" OR knickerbocker) AND aortic dissection | 58 |
| 9 | TEVAR AND (subacute OR timing) AND "type B" AND (remodeling OR remodelling) | 38 |
| 10 | TEVAR AND "spinal cord ischemia" AND (coverage OR "extent of coverage" OR celiac) | 10 |
| 11 | "stent graft-induced new entry" AND (meta-analysis OR "systematic review") | 3 |
| 12 | (Marfan OR "connective tissue disease" OR "heritable thoracic aortic disease") AND (TEVAR OR frozen elephant trunk) | 25 |
| 13 | ("branched arch" OR "arch branch" OR NEXUS OR "endovascular arch repair") AND dissection | 109 |
| 14 | (staged OR staging) AND "spinal cord" AND (thoracoabdominal OR "segmental artery") | 44 |
| 15 | frozen elephant trunk AND ("bare stent" OR "bare metal stent" OR "uncovered stent") | 16 |
| 16 | frozen elephant trunk AND thoracoabdominal AND (open repair OR "third stage") | 34 |
| 17 | aortic dissection AND ("4D flow" OR "false lumen ejection" OR "false lumen pressure") | 42 |
| 18 | aortic dissection AND ("deep learning" OR "artificial intelligence") AND (remodeling OR remodelling OR "false lumen") | 12 |
| 19 | frozen elephant trunk AND TEVAR AND (review OR "meta-analysis") | 82 |
| 20 | Targeted searches for guidelines, consensus documents, reporting standards, and randomized trials (EACTS/STS 2024, ESVS 2026, ACC/AHA 2022, SVS/STS reporting standards, STORAGE, ARCH-ARC, INSTEAD-XL, ADSORB, ERADICARE, PAPAartis, trial registration of early endovascular repair after frozen elephant trunk) | Not counted |

Records: number of PubMed records returned on the search date. Search 20 comprised targeted searches by title, trial acronym, or document name; these records were not counted. ACC/AHA: American

College of Cardiology/American Heart Association; ARCH-ARC: ARCH-Academic Research Consortium; EACTS: European Association for Cardio-Thoracic Surgery; ESVS: European Society for Vascular Surgery; STS: Society of Thoracic Surgeons; SVS: Society for Vascular Surgery; TEVAR: thoracic endovascular aortic repair.
